# A randomized controlled study of 5 and 10 days treatment with phenoxymethylpenicillin for pharyngotonsillitis caused by streptococcus group A – a protocol study

**DOI:** 10.1186/s12879-016-1813-7

**Published:** 2016-09-13

**Authors:** Gunilla Skoog, Charlotta Edlund, Christian G. Giske, Sigvard Mölstad, Christer Norman, Pär-Daniel Sundvall, Katarina Hedin

**Affiliations:** 1 Unit for Antibiotics and Infection Control, The Public Health Agency of Sweden, Solna, Sweden; 2 Department of Medicine Solna, Division of Infectious Diseases, Karolinska Institute, Stockholm, Sweden; 3 Division of Clinical Microbiology, Department of Laboratory Medicine, Karolinska Institutet, Stockholm, Sweden; 4 Department of Clinical Microbiology, Karolinska University Hospital, Stockholm, Sweden; 5 Department of Clinical Sciences, Malmö, Family Medicine, Lund University, Lund, Sweden; 6 Salem Primary Health Care Center (PHCC), Säbytorgsvägen 6, SE-144 30 Rönninge, Sweden; 7 Närhälsan Research and Development Primary Health Care, Region Västra Götaland, R & D Center Södra Älvsborg, Sven Eriksonsplatsen 4, SE-503 38 Borås, Sweden; 8 Department of Public Health and Community Medicine/Primary Health Care, Institute of Medicine, Sahlgrenska Academy at the University of Gothenburg, Box 454, SE-405 30 Göteborg, Sweden; 9 Department of Research and Development, Region Kronoberg, Växjö Sweden

**Keywords:** Pharyngotonsillitis, Treatment duration, Phenoxymethylpenicillin, Penicillin V, RCT, Primary health care, Streptococcus, GAS, Centor criteria

## Abstract

**Background:**

In 2014 the Swedish government assigned to The Public Health Agency of Sweden to conduct studies to evaluate optimal use of existing antibiotic agents. The aim is to optimize drug use and dosing regimens to improve the clinical efficacy. The present study was selected following a structured prioritizing process by independent experts.

**Methods:**

This phase IV study is a randomized, open-label, multicenter study with non-inferiority design regarding the therapeutic use of penicillin V with two parallel groups. The overall aim is to study if the total exposure with penicillin V can be reduced from 1000 mg three times daily for 10 days to 800 mg four times daily for 5 days when treating *Streptococcus pyogenes* (Lancefield group A) pharyngotonsillitis. Patients will be recruited from 17 primary health care centers in Sweden. Adult men and women, youth and children ≥6 years of age who consult for sore throat and is judged to have a pharyngotonsillitis, with 3–4 Centor criteria and a positive rapid test for group A streptococci, will be included in the study. The primary outcome is clinical cure 5–7 days after discontinuation of antibiotic treatment. Follow-up controls will be done by telephone after 1 and 3 months. Throat symptoms, potential relapses and complications will be monitored, as well as adverse events. Patients (*n* = 432) will be included during 2 years.

**Discussion:**

In the era of increasing antimicrobial resistance and the shortage of new antimicrobial agents it is necessary to revisit optimal usage of old antibiotics. Old antimicrobial drugs are often associated with inadequate knowledge on pharmacokinetics and pharmacodynamics and lack of optimized dosing regimens based on randomized controlled clinical trials. If a shorter and more potent treatment regimen is shown to be equivalent with the normal 10 day regimen this can imply great advantages for both patients (adherence, adverse events, resistance) and the community (resistance, drug costs).

**Trial registration:**

EudraCT number 2015-001752-30. Protocol FoHM/Tonsillit2015 date 22 June 2015, version 2. Approved by MPA of Sweden 3 July 2015, Approved by Regional Ethical Review Board in Lund, 25 June 2015.

## Background

In 2014 the Swedish government assigned to The Public Health Agency of Sweden to conduct studies to evaluate existing antibiotic agents from new perspectives. The aim is to optimise drug use and dosage regimens to improve the clinical efficacy of existing antibiotics, and to reduce ecological disturbances in the normal microbiota. The clinical trial described below is one of the projects prioritized by independent experts within this assignment.

Pharyngotonsillitis is one of the most common infections in primary health care (PHC) in Sweden [1] and accounts for about 20 % of all antibiotic prescriptions within PHC [2]. The estimated number of patients in Sweden treated for pharyngotonsillitis is about 400,000 each year [3]. Streptococcus group A (GAS) is the most common bacterial etiology and also the dominating reason for antibiotic treatment [3]. According to the Swedish guidelines the recommendation is to treat pharyngotonsillitis with phenoxymethylpenicillin (penicillin V) if 3–4 Centor criteria [2] and GAS is present [3]. The recommended dose for adults is 1000 mg penicillin V three times daily for 10 days which corresponds to about 12 t of penicillin V each year in Sweden, calculated on the basis of adult doses. The infection is most frequent among school children and young adults, resulting in a somewhat lower total amount of consumption [4].

According to present European and American guidelines, 10 days penicillin treatment is generally recommended as first line treatment of streptococcal pharyngotonsillitis [5, 6]. The historical reason for 10 days treatment duration with penicillin is mainly to avoid serious complications like acute rheumatic fever and glomerulonephritis. These conditions are currently extremely rare in western countries [7]. According to a Cochrane report from 2012 patients recover without antibiotic therapy but treatment is sometimes advised to hasten clinical resolution and prevent suppurative sequelae, like peritonsillitis, acute otitis media or sinusitis. Clinical studies on shorter treatment duration with penicillin for streptococcal pharyngotonsillitis are encouraged [8].

There are few published studies comparing 10 days treatment duration with shorter treatment durations with penicillin V. Three studies which were published in the 1980s have been identified. Five versus 10 days (250 mg three times daily and 800 mg twice daily respectively) [9, 10] and 7 versus 10 days (500 mg three times daily) [11] were studied. All of the studies concluded that 10 days of treatment was preferable but in the light of current knowledge of pharmacokinetics and pharmacodynamics (PK/PD) the doses used in these studies were suboptimal. The effect of beta-lactam antibiotics is dependent on time above the minimum inhibitory concentration (MIC) of the unbound drug concentration in serum (free drug time above MIC; fT > MIC). Usually, the target is that 95–99 % of the population should achieve a fT > MIC of around 30–40 % for non-severe infections [12], also expressed as the population target attainment for a given target. For beta-lactams a more frequent dosing is preferred and the dosing interval is more critical than the size of the dose [12]. The dosing regimen 800 mg four times daily provides better target attainment compared to 1000 mg three times daily (33 % fT > MIC compared to 25 % fT > MIC at the breakpoint MIC 0.25 mg/L). Our hypothesis is that the experimental dosage regimen 800 mg four times daily gives at least the same efficacy, possibly better patient adherence and a significantly lower total exposure.

In the present study we will examine if penicillin V given four times daily for 5 days is non-inferior to the currently recommended dose regimen of 10 days to patients with pharyngotonsillitis caused by streptococcus group A. Reducing the treatment duration to 5 days will almost halve the consumption of penicillin V for this indication. A shorter treatment duration may potentially improve patient adherence [13], cause less effect on the human microbiota [14] as well as lower medicinal costs for patients and community.

## Methods

### Aim

The overall aim is to study if the total exposure with antibiotics can be reduced from 1000 mg three times daily for 10 days to 800 mg four times daily for 5 days when treating streptococcus group A pharyngotonsillitis with penicillin V.

### Design

This Phase IV study is a randomized, open-label, multicenter study with non-inferiority design regarding the therapeutic use of penicillin V with two parallel groups.

### Setting

Patients will be recruited from 17 primary health care centers (PHCC) in the counties of Skåne, Kronoberg and Västra Götaland in the southern of Sweden. The PHCCs are representing both urban and rural regions.

### Characteristics of the patients

#### Inclusion criteria

Adult men and women, youth and children ≥6 years of age who are seeking a PHCC for sore throat with suspected tonsillitis and meeting the criteria in accordance with current treatment recommendations for streptococcal pharyngotonsillitis, i.e. 3–4 Centor criteria (fever ≥ 38.5, tender lymph nodes, coatings of the tonsils and absence of cough) and a positive rapid antigen detection test (RADT) for GAS will be included in the study [15].

#### Exclusion criteria

A patient is not eligible for inclusion if any of the following criteria apply: 1) Signs of serious illness according to pre-specified criteria, 2) hypersensitivity to penicillin, 3) chronic illness that significantly impair the immune system, 4) immunomodulating treatment, 5) treatment with antibiotics for pharyngotonsillitis during the last month (relapse), 6) any antibiotic treatment during the last 72 h before inclusion, 7) difficulty swallowing tablets, 8) any other cause which, according to the physician’s judgment, makes it unsuitable for the patient to participate in the study.

#### Intervention

Patients will be randomized to treatment with penicillin V four times daily for 5 days or three times daily for 10 days. The dosages for different weight groups are presented in Table 1.

**Table 1:** Penicillin V dosing schedule

Body weight	Dosage 5 days treatment	Dosage 10 days treatment
>40 kg	800 mg × 4	1000 mg × 3
20–40 kg	500 mg × 4	500 mg × 3
10–20 kg	250 mg × 4	250 mg × 3

#### Randomization

Randomization within blocks for each PHCC separately was performed by a statistician at The Public Health Agency of Sweden. Sealed envelopes with unique numbers and allocated treatment were distributed to each of the 17 participating PHCCs.

### Context and procedure

Study procedures are presented in Table 2.

**Table 2:** Study procedures for both intervention groups, penicillin V given four times daily for 5 days or three times daily for 10 days

	Baseline	5–7 days after penicillin V treatment discontinuation.	1 month (by telephone)	3 months (by telephone)
Clinical judgement by physician	X	X		
Anamnesis	X			
Written informed consent	X			
Inclusion/exclusion criteria	X			
Concomitant drug treatment	X	X		
Rapid antigen detection test (RADT)	X	X		
Throat swab culture	X	X		
Evaluation of primary endpoint		X		
Monitoring adverse events		X	X^a^	
Monitoring relapses		X	X	
Monitoring complications			X	X

^a^Follow-up of adverse events which were ongoing at previous contact

#### Visit one

Patients presenting with sore throat will be clinically examined by a physician. In case of suspected faryngotonsillitis and 3–4 Centor criteria a RADT for GAS will be performed. Patients with a positive RADT test for GAS who meet all the inclusion criteria, and none of the exclusion criteria, will be asked to participate in the trial. After signed informed consent, by patient or guardian, the patient will be asked about medical history, demographic data, previous antibiotic exposure and concomitant drug treatment.

A throat swab for semi quantitative cultures of streptococcus group A, C and G will be performed.

After randomization to intervention group 5 or 10 days the physician prescribes penicillin V which the patient will retrieve from the pharmacy. A patient diary is handed out which the patient or guardian is asked to fill in until the follow-up visit, five to seven days after treatment discontinuation.

Demographic data and clinical data will be recorded in a case report form (CRF) by study personnel. In addition, data regarding intake of penicillin V doses and analgesics, presence of sore throat and fever, adverse events etc. is filled in daily by each patient or guardian in the diary.

#### Test-of-cure visit

A test-of cure (TOC) visit will be performed at the PHCC 5 to 7 days after completion of antibiotic treatment. Clinical judgement of throat status, a new RADT and culture, and recording of adverse events will be performed.

#### Follow-up controls

Follow-up controls will be done by a telephone call 1 month (28–35 days) and 3 months after the last treatment day. Throat symptoms, potential relapses and complications will be monitored as well as adverse events ongoing at previous visits.

### Outcomes

#### Primary outcomes

The primary outcome is clinical cure 5–7 days after discontinuation of antibiotic treatment (TOC). The patients will be asked about throat symptoms and a clinical judgement will be performed by the investigator. Clinical cure is defined as complete recovery without remaining/residual symptoms or clinical findings of pharyngotonsillitis or symptomatic relapse at TOC [15].

#### Secondary outcomes

The secondary outcomes are bacteriological eradication according to the culture taken at the TOC, time to relief of symptoms according to the patient diary, frequency of relapses one month after first diagnosis, frequency of complications during the study period as well as pattern of adverse events.

### Definitions

Definitions of outcome measures are presented in Table 3.

**Table 3:** Definitions of outcome measures

Term	Definition
Clinical cure	Clinical judgement by physician 5–7 days after discontinuation of treatment. Patient completely recovered with no remaining/residual symptoms or clinical findings of pharyngotonsillitis or symptomatic relapse at TOC.
Therapeutic failure	None or inadequate clinical effect during treatment period.
Not evaluable for primary outcome	Follow-up after discontinuation of treatment is missing.
Relapse	Symptomatic pharyngotonsillitis within one month after first diagnosis, according to clinical judgement by physician 1) antibiotic treatment necessary or 2) verified pharyngotonsillitis caused by streptococcus. Recurrent symptomatic pharyngotonsillitis presenting before and including the TOC visit, following at least 1 day without symptoms after initial treatment, will be judged as early relapse.
Complication with suspected relation to the primary infection	For example peritonsillitis, acute otitis media or sinusitis during the study period, i.e. until the last follow-up visit.
Bacteriological cure	Absence of GAS in culture. If culture is missing, a negative RADT.
Asymptomatic carriage of GAS	Presence of GAS in culture from patient *without* throat symptoms. If culture is missing, a positive RADT.
Symptomatic infection of GAS	Presence of GAS in culture from patient *with* throat symptoms. If culture is missing, a positive RADT.

### Statistical analysis

#### Sample size/power calculation

The power calculation is based on the assumption of 90 % clinical recovery in the reference group and no difference between the two groups. The non-inferiority limit is set to minus 10%, the confidence level (two-sided) to 95 %, the power to 85 % and the distribution between the groups to 1/1. Based on these presumptions, 324 evaluable patients (162 in each group) are needed to be able to demonstrate non-inferiority between treatment groups. Presuming that 25 % of the included patients will not be possible to evaluate 432 (324*(1/0.75)) patients are needed to be included.

All effect variables will be analyzed for per protocol population (PP) as well as for the modified intention to treat (MITT) population as sensitivity analysis.

#### Safety

Patterns of adverse events between the two treatment groups will be evaluated and analysed descriptively. Active surveillance of adverse events will be done by a physician at TOC and by self-reporting by the patient/guardian in the patient diary. At the follow-up controls 1 and 3 months after discontinuation of treatment, patients will be asked about adverse events that are still ongoing at the previous visit.

#### Monitoring

Monitoring according to GCP/ICH will be performed by trained personnel allocated by the sponsor, The Public Health Agency of Sweden.

#### Ethical issues

It is important to include children in clinical trials, especially since children often suffer from infections and therefore are treated with antibiotics more often than other age groups. The lower age limit 6 years was chosen based on presumed ability to swallow tablets, since exposure is easier to control with tablets than with oral suspension.

We consider that there are no risks to participate in the study, regardless of age. The sampling is simple and follow the normal routines at the PHCCs. Both treatment alternatives are expected to be effective against the infection. A shorter treatment regimen could include benefits in form of less adverse events and less disturbances in the human microbiota. In addition, patient adherence to the treatment may improve with shorter treatment duration.

## Discussion

In the era of increasing antimicrobial resistance and the shortage of new antimicrobial agents it is necessary to revisit old antibiotics to ensure that they are used correctly and to their full potential. Important old drugs deserve to be rigorously tested according to today’s standards and requirements for clinical studies. Old antimicrobial drugs are often associated with inadequate knowledge on PK/PD and lack of optimized dosing regimens based on randomized controlled clinical trials.

Since old antibiotics are usually out of patent protection, financial incentives to perform the necessary studies are lacking. Thus, public funding is often the only option available [16]. For the current study, funded by the Swedish government, an initial systematic review to identify knowledge gaps was performed, followed by a structured prioritizing process by independent experts, in order to select the most needed non-clinical and clinical studies.

There is currently no substantial scientific evidence to recommend less than 10 days treatment of penicillin V against streptococcal pharyngotonsillitis. A shorter treatment period though, with lower total antibiotic exposure, is expected to reduce the risk of ecological disturbance on the human microbiota [17] and possibly improve patient adherence [13].

One possible disadvantage with a shorter treatment duration could be an increased risk for relapses, since the time with protection against a new infection is shorter. Furthermore, it could be argued that the daily dose for the 5-day treatment is slightly higher than the recommended daily dose, which may increase the risk for adverse events. However, there is extensive clinical experience of even higher doses in treatment of other infections like acute otitis media, sinusitis, and dental infection, justifying the safety profile of penicillin V also at high doses.

Overall we believe that this study, facilitated by public funding, may contribute with important knowledge and may have a substantial impact on public health. If a shorter and more potent treatment regimen is shown to be equivalent with the normal 10 day regimen this can imply great advantages for both patients (resistance, adverse events, adherence) and the community (resistance, drug costs).

## Abbreviations

CRF: Case record form
GAS: Gtreptococcus group A
GCP: Good clinical practice
ICH: International conference on harmonization
MIC: Minimum inhibitory concentration
MITT: Modified intention to treat population. Patients who received at least one dose of the study drug
PHC: Primary health care
PHCC: Primary health care centre
PK/PD: Pharmacokinetics and pharmacodynamics
PP: Per protocol population. Patients without violations to the study protocol, receiving at least 80 % of the prescribed study drug and evaluable at least until the TOC visit.
RADT: Rapid antigen detection test
RCT: Randomized controlled trial
TOC: Test of cure
